# 1410. Improved Disinfection in the Operating Room through Hydrogen Peroxide Fogging

**DOI:** 10.1093/ofid/ofad500.1247

**Published:** 2023-11-27

**Authors:** Jennifer Davis, Tina Bair, Brian Lapolla, Michael Bigham

**Affiliations:** Akron Children's Hospital, Akron, Ohio; Akron Children's Hospital, Akron, Ohio; Akron Children's Hospital, Akron, Ohio; Akron Children's Hospital, Akron, Ohio

## Abstract

**Background:**

Hospital acquired infections (HAIs), including surgical site infections (SSIs), are a major contributor to morbidity. Environmental exposure is a contributing factor to HAIs. Environmental decontamination with hydrogen peroxide fogging has proven to reduce bacterial growth and bioburden in the hospital setting. We sought to 1) implement and test feasibility of an operating room (OR)-based hybrid hydrogen peroxide fogging program and 2) assess the impact of hydrogen peroxide fogging on SSIs.

**Methods:**

At a large freestanding midwestern Children's Hospital with a surgical volume of over 17,000 annual surgeries, a hybrid hydrogen peroxide fogging program was implemented in a risk-stratified fashion. Baseline fogging was completed to establish a post-fogging time series for bacterial regrowth (measured by culture and bioburden) and to guide the implementation schedule. Based on time-series results, room-specific fogging parameters were established. ORs received fogging either every 2 weeks (neurosurgical, cardiac and spine ORs) or every 4 weeks (all other ORs).

The fogging program was administered using a third-party vendor beginning in September 2022. The feasibility assessment was designed to identify any safety events, interruptions in care or access to the ORs due to fogging. The time series outcomes were bioburden and bacterial cultures. The primary patient outcome measure was the rate of SSIs. This is depicted in both a P chart, showing a monthly rate, and a T-chart, showing days between events. SSIs for orthopedic, spine and neurosurgeries were tracked from January 2021 to present.

**Results:**

After implementation of the program there was a significant decrease in SSI rates from 3.38% to 0.68% and is represented by a center-line shift in the P chart. The days between SSIs moved in a favorable direction, increasing to 238 days, the longest since January 2021. The time series evaluation indicated return to baseline bacterial bioburden between 3 and 4 weeks post-fogging. The feasibility assessment identified no safety events, delays, interruptions or decreases in OR utilization.

Surgical Site Infections (Spine, Cardiac, Neurosurgical)

**Figure:**
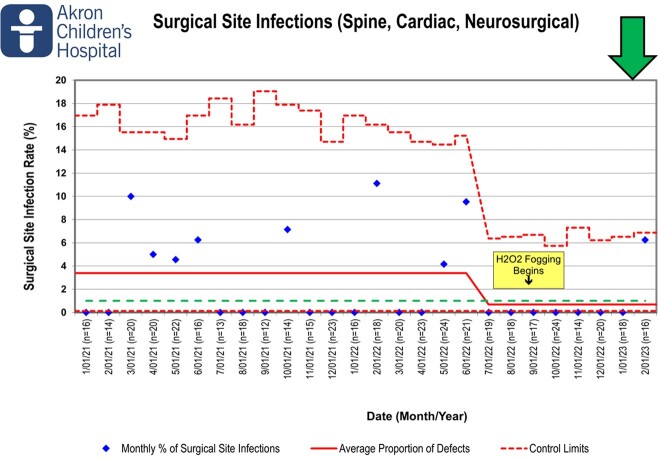

Since fogging began in September of 2022, we have experienced a centerline shift demonstrating a reduced rate of surgical site infections from 3.38% to 0.68%, the longest stretch between surgical site infections since January 2021.

Days Between Surgical Site Infections

**Figure:**
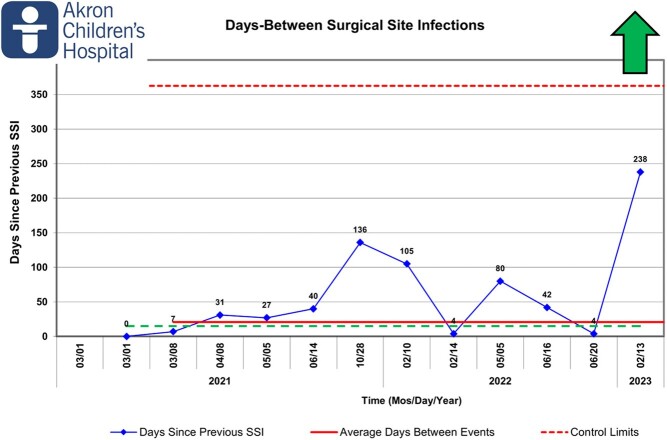

Since fogging began in September of 2022, we have experienced the longest stretch between surgical site infections since January 2021

**Conclusion:**

Hydrogen peroxide fogging is a feasible form of disinfection and reduces SSI rates when applied in the OR of a Children’s Hospital with a moderate to large surgical volume.

**Disclosures:**

**Michael Bigham, MD**, ForTec Medical: Advisor/Consultant

